# Supplementary figures and images for: Functional Suppression of CLOCK Activity in Ventromedial Hypothalamic Prodynorphin Neurons Alters Locomotor Activity and Rapid Eye Movement Sleep
Source: Neurol Int. 2025 Dec 25;18(1):5. doi: 10.3390/neurolint18010005 (PMC12844858; doi:10.3390/neurolint18010005)

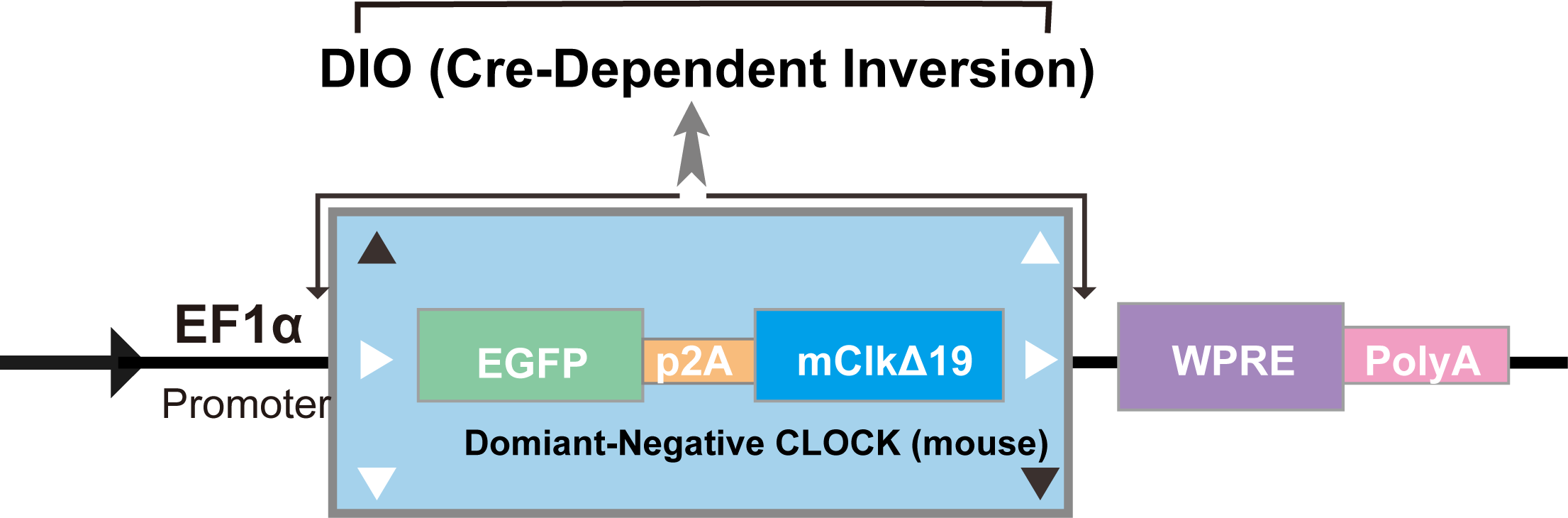

Supplement: Supplementary file 1 [file neurolint-18-00005-s001.zip › Supplementary Figure S1.tif]
